# The bridging and bonding structures of place-centric networks: Evidence from a developing country

**DOI:** 10.1371/journal.pone.0221148

**Published:** 2019-09-05

**Authors:** Didem Gündoğdu, Pietro Panzarasa, Nuria Oliver, Bruno Lepri

**Affiliations:** 1 Department of Information Engineering and Computer Science, University of Trento, Trento, Italy; 2 Center for Information Technology, Fondazione Bruno Kessler, Trento, Italy; 3 Queen Mary University of London, London, United Kingdom; 4 Data-Pop Alliance, New York, United States of America; Universitat de Barcelona, SPAIN

## Abstract

Social capital has long been associated with opportunities of access to valuable resources that individuals, groups, communities, and places can extract from the social structure emerging from their interactions. Despite the overall consensus on the structural signature of social capital, there is still controversy over the relative benefits associated with different types of social structure. In this article, we advocate a two-faceted perspective on social capital, regarded as value originating from both closed (rich in third-party relationships) and open (rich in brokerage opportunities) bridging structures. We uncover these structures from place-centric networks and investigate their association with key socio-economic indicators. To this end, we draw on aggregated mobile phone usage data, and construct the place-centric communication and mobility networks in the city of Abidjan in Côte d’Ivoire. We begin by defining appropriate network metrics to capture the interplay between bonding and bridging social structures in each of the 10 districts (communes) in Abidjan. We then examine the correlation between these metrics and a number of district-level socio-economic indicators related to economic prosperity, wealth, security and democratic participation. Our findings suggest that both closed and open structures can serve as wellsprings of social capital: while closed bonding structures are associated with higher economic prosperity, open bridging structures are associated with increased democratic participation and security. By uncovering sources of social capital from communication and mobility place-centric networks in a developing country, our work contributes to a better understanding of how these networks could be used to enhance and sustain socio-economic growth and prosperity, and ultimately paves the way towards a broader comparative study of social capital in developed and developing countries.

## Introduction

Over the last few decades, social capital has been studied across various fields, including sociology [1–4], economics [5], and political sciences [6]. There is agreement on the salience of social structure for promoting the economic prosperity and the development of modern societies. In particular, previous work has highlighted the role played by social capital for economic growth [7], and has suggested that it can reduce the transaction costs associated with formal coordination mechanisms [8], and can nurture strong economic performance and financial development [9]. Moreover, patterns of social interactions and structures have been associated with additional direct and indirect benefits, such as helping people in their search for new job opportunities [10, 11], creating new businesses [12, 13], enabling safe and secure residential environments [14], improving public health [15–17], increasing democratic participation [2, 18], and reducing corruption [19, 20].

Social capital can be defined as the value that individuals, groups, communities or places can gain as a result of the “investment in social relations, bonding similar people together and bridging diverse people, with norms of reciprocity” [21, 22]. From this perspective, it is clear that social capital refers to value that can be generated through networks of interacting nodes (*e.g*., individuals, places). In this sense, scholars across the social sciences converge in emphasizing the salience of social structure for social capital [23].

However, in the literature there are still controversies and debates on the specific types of social structure that matter [24]. In particular, social capital does not have a unique and undisputed network-based definition, and scholars tend to vacillate between two diverging positions, each emphasizing a specific structure and network mechanism regarded as the main determinants of social capital: *closed bonding structures and social cohesion* on the one hand, and *open bridging structures and brokerage opportunities* on the other.

- *Closed bonding structures* produce social capital through the generative mechanism of social cohesion, leading any two nodes in the network that share a neighbour to also establish a connection between themselves. Closure of open triads eventually sustains trust, promotes cooperation, and nurtures the development of a common social identity [1].
- *Open bridging structures* generate value through structural cleavages separating individuals, groups, or places, and through opportunities for intermediating between otherwise disconnected parts of the network [4, 10]. Through weak ties and brokerage opportunities, open structures elicit value by enabling nodes to leverage the diversity of their contacts, including diversity in demographic characteristics, socio-economic levels, cultural background. Most importantly, by spanning holes in the network nodes can recombine various sources of ideas and information, thus sustaining the generation of new knowledge and innovation [4, 25].

In addition, previous studies have analyzed two different levels of aggregation in their efforts to assess social capital: the *individual* and *collective* levels [23]. At the individual level, social capital refers to the benefits that an individual can derive by engaging in various patterns of social interaction. At the collective level, social capital captures the benefits of a higher-level unit of analysis such as a group, a community, a place or a country [26].

While the importance of social capital has been widely established in the literature [27–29], measuring how social capital is generated and how individuals, groups or places can secure access to it is a difficult task posing a number of methodological challenges. In the sociology and economics literature, three main approaches are used to measure social capital, based on: (i) surveys [6], (ii) data on non-profit associations and selfless activities, such as donating blood or voting in a referendum [30]; and (iii) experimental in-lab settings [31].

In this article, we propose to use mobile phone data to construct network variables and measure both bonding and bridging social structures. We focus on *collective social capital* at a district (commune) level in a large city of a developing African country (*i.e*., Abidjan, the economic capital of Côte d’Ivoire). In addition, we analyze the relationship between the proposed mobile phone data-based network metrics and a number of socio-economic indicators. To the best of our knowledge, this is the first empirical work to draw on mobile network data to investigate the interplay between open and closed social structures and their roles as vehicles of collective social capital in a developing economy.

Using mobile phone data to detect social capital has important advantages over previous approaches. In particular, it enables us to draw on real-world data rather than experimental data collected in the lab. It also enables us to construct a large-scale data set, given the widespread adoption of mobile phones both in developed and developing economies. Finally, it offers a methodological framework that could easily be extended and generalized to other locations and countries [32].

While mobile phone data could offer significant advantages as a relevant source of information on the social capital within a region or country, relatively little research has been carried out on the interplay between bridging and bonding social structures at that level of analysis [33]. Moreover, the structural foundations of social capital within a developing economy are still largely to be investigated. This article aims to take a step in this direction, and in so doing it engages with current debates on the trade-off between various network sources of social capital [24].

Using mobile phone data, we construct two place-centric networks, a communication and a mobility network, to uncover the mechanisms through which these two networks can serve as wellsprings of social capital at the district level. In particular, we aim to shed light on the role played by *bridging* and *bonding* structures of place-centric networks in fostering economic well-being, safety, and democratic participation. We provide empirical evidence on the existence of different structural sources and generative mechanisms of social capital across a large city’s different districts, and show how the association between structure and value creation depends on the types of valuable outcomes under evaluation (*e.g*., economic prosperity, civic engagement or security).

It is worth emphasizing that the goal of our work is not to study sources of social capital at the individual level. Indeed, we do not aim to investigate whether individuals can benefit from the way they communicate and move across places. By contrast, here we focus on the topological positions occupied by geographical places—the communes—within the wider network of communication and mobility flows characterizing a city (Abidjan). We then examine the extent to which these places can leverage their positions in the underlying network to enhance value creation. While our focus is on places and not individuals, nonetheless the social capital that communes secure from their network can ultimately benefit all individuals that, for a wide range of reasons, are associated with these places (e.g., residents, workers, passers-by).

In sum, our work contributes to a better understanding of the intricate relationship between place-centric communication and mobility networks in a developing country, and sheds a new light on the association between these networks, socio-economic growth and prosperity.

## Related work

In recent years, there have been a number of attempts to conduct urban analysis using mobile phone data [34–42]. For example, a common application of mobile phone data has been concerned with the estimation of the population density in different locations [43]. This application is very useful when the focus of the analysis is a developing country, where census data is typically extremely difficult to obtain and existing databases are often obsolete.

Other studies have investigated the relationship between geography and communication patterns, providing evidence that the distance between cities has a negative association with the intensity of communication between them [44, 45]. Related studies, instead of focusing on communication between cities, have analyzed the relationship between the size of a city and the structure of the communication networks of its residents [46]. For example, it has been shown that the number of contacts and the volume of communication activity grow with city size, while the probability of befriending a friend’s friend is independent of city size [46]. Similarly, other studies have drawn social borders and uncovered communities within a country or a city based on the communication and interactions between geographical locations (*e.g*., communes, counties, neighborhoods, venues) [35, 47, 48].

Another line of work has investigated the usage of fixed landline and/or mobile phone data to evaluate the socio-economic characteristics of the different regions of a country. A seminal study in this direction has been the one conducted by Eagle *et al*. based on data from the United Kingdom [49]. More specifically, the authors have shown that the social and geographical diversity of an individual’s contacts (measured through the entropy of the individual’s neighborhood) correlates positively with the socio-economic conditions of the area in which the individual lives.

In a more recent study, Mao *et al*. have proposed the *CallRank* metric, which was obtained by applying a weighted PageRank algorithm to the aggregated mobile phone network of Côte d’Ivoire [50]. Interestingly, it has been suggested that high values of the CallRank metric are associated with regions that are vital for the economy of the country. Using the same data set, Smith-Clarke *et al*. have focused on a number of properties of communication, and then evaluated their correlation with poverty levels [51]. In particular, it has been found that poverty levels are associated with deviations from the expected flow of communication: that is, if the amount of communication from and to a certain geographical area is significantly lower than expected, then the area is likely to suffer from high poverty levels. It is worth noting that both Mao *et al*. [50] and Smith-Clarke *et al*. [51] have constructed an aggregated mobile phone network, where the nodes are the cell towers and the weight of a link between two cell towers refers to the number of calls made between them. Our work dovetails with these two studies in constructing and examining place-centric communication and mobility networks within the city of Abidjan. In addition, our work complements and extends these studies by analyzing the association between the bonding and bridging structures of the communication and mobility networks on the one hand, and the economic prosperity, civic engagement and social security of the communes in Abidjan on the other.

## Material and methods

Mobile telecommunication operators collect Call Detail Records (CDRs) for billing purposes. These records generally consist of a number of fields, including the encrypted originating and destination numbers, the timestamp of the call, the duration of the call and the location of the cell towers the originating number was connected to (the location of the destination number’s cell tower is only available if the destination number belongs to the same operator). Typically, there are CDRs for voice, SMS and data traffic events. Even though the data is usually encrypted and all personal information is removed, CDRs are not easily accessible as a result of regulatory and legal constraints. The data sets we analyze in this article were provided as part of the Data For Development (D4D) Challenge 2013 organized by Orange Telecom and the MIT Media Lab [52].

### D4D Côte d’Ivoire data set

The data set contains anonymized and aggregated calls between 5 million customers from December 1, 2011 to April 28, 2012. Specifically, the data set contains the total volume (number of calls and SMSs) and the duration of calls between each pair of cell towers over the entire time period. The total number of cell towers is 1, 238. The exact locations of the towers were not provided by Orange as a result of the company’s operational confidentiality. However, they provided an approximate *latitude* and *longitude* location of each cell tower (obtained by adding noise to the actual location). The data consists of the hourly aggregated total traffic volume between cell towers, and hence no individual data is shared or analyzed.

The widespread usage of mobile phones across Côte d’Ivoire (95%) [53] ensures that our study is sufficiently representative of the whole population. Moreover, the network operator holds a dominant position with 48% of the market share. The details of the data corpus of D4D are provided in Table 1. While the D4D Challenge shared 4 subsets of data, we focused our analysis on two of them:

1. Subset-1, containing hourly aggregated data about the communication between the operator’s cell towers, provides for each entry: (i) date, (ii) hour, (iii) initiating cell tower id, (iv) receiver cell tower id, (v) aggregated number of calls, and (vi) aggregated duration of calls;
2. Subset-2, containing information on customers’ behavior, provides for each entry: (i) anonymized user id, (ii) date of call, (iii) hour of call, and (iv) initiating cell tower id.

**Table 1:** Data summary of D4D subsets.

	Subset-1	Subset-2	Subset-3	Subset-4
**No. users**	-	50,000	500,000	5,000
**Duration**	5 months	5 months	5 months	5 months
**Type**	Antenna-Antenna Call	User-Antenna	User Subpref.	User-User

We use Subset-1 to construct the place-centric communication network and Subset-2 to construct the place-centric mobility network. We build both networks at a city (Abidjan) and regional (sub-prefectures) levels. From these two networks, we compute several metrics that characterize sources of collective social capital. Using these metrics we investigate the topological role played by a geographical place (*i.e*., the commune) in the networks of communication and mobility flows. Following the recent works by Smith-Clarke *et al*. [51] and Mao *et al*. [50], we focus on the higher-level unit of the cell tower, and thus assume that the structure of the connections between cell towers can reasonably reflect the structure of the connections between the places and the communities of individuals associated with the corresponding locations of the cell towers. For example, a cell tower with a high value of betweenness centrality (or effective size) is likely to refer to a place that plays a crucial intermediary role in the network. This place and its associated community of people may gain a considerable influence over other locations and their associated communities, as a result of their control over communication and mobility between otherwise disconnected parts of the network. Drawing on the network between cell towers (and associated locations), we then investigate the association between the structural positions of locations in such network and a number of publicly available socio-economic indicators related to their economic well-being, civic participation, and security levels. Since these socio-economic indicators are more detailed at the city level than at the sub-prefecture level, we opt to restrict our analysis of social capital to Abidjan, the largest city in Côte d’Ivoire. In our study the communities associated with the location of each cell tower include not only the residents but also the people that work, study, or simply spend some time in the location. Indeed, several studies have suggested that to properly examine the efficient allocation of public resources and service provision to a given area (based, for example, on its security level and risk of victimization), it is important to monitor not only the resident population but also the ambient population, namely the people that are present in the area at any given time [54, 55].

#### Abidjan, the economic capital of Côte d’Ivoire

**Fig 1:**
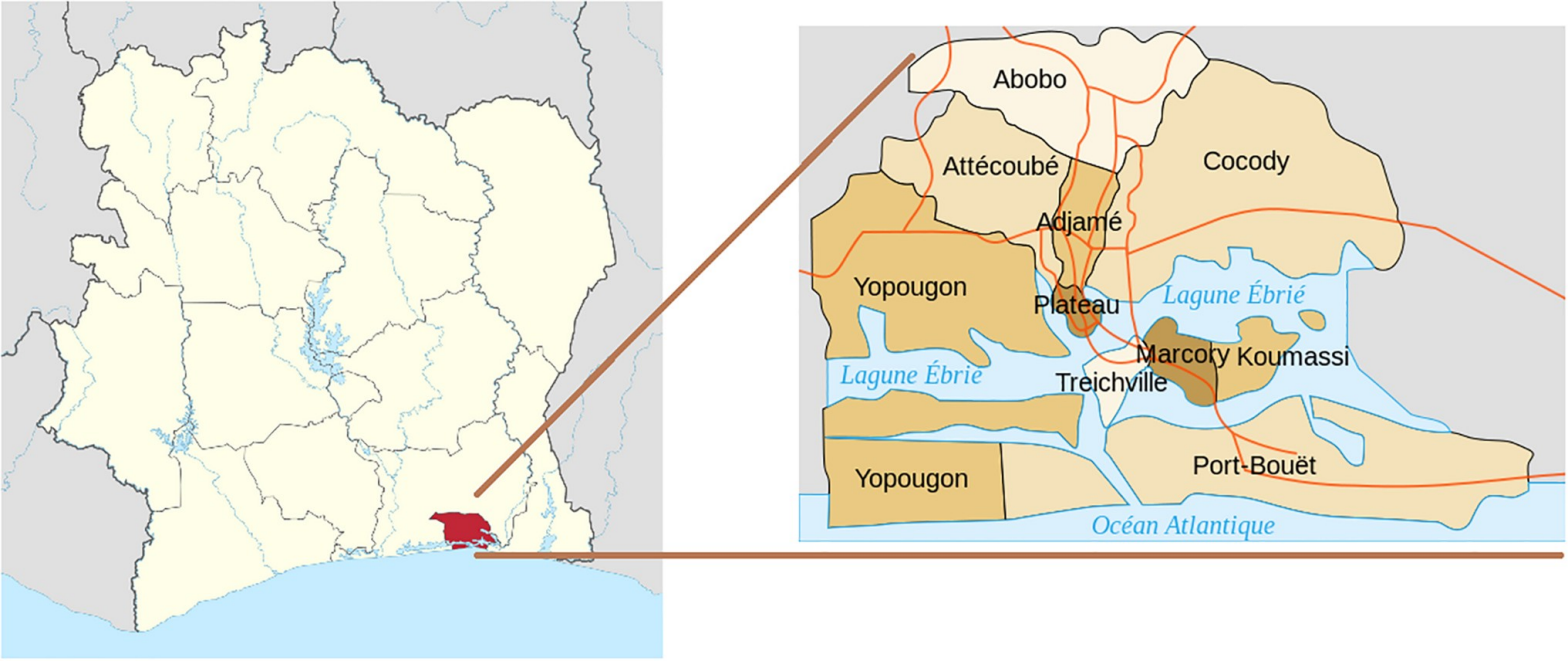
Map of 10 communes of Abidjan. Left: Abidjan, the Côte d’Ivoire’s economic capital, shown in red. Right: 10 communes of Abidjan.

Abidjan is the most populated city in Côte d’Ivoire, with 20% of the country’s total population. It is composed of 10 communes, as shown in Fig 1 and in Table 2. Given its population and economic power, it is the region in the country with the densest levels of mobile cell towers (antennae), with 378 cell towers out of 1, 238.

**Table 2:** Population, area coverage in *Km*^2^, and number of cell towers in each commune of Abidjan. Data are obtained from the 2014 census.

Commune	Population	Area (Km^2^)	Number of cell towers
Abobo	1,030,658	112.7	45
Adjame	372,978	12.1	23
Attecoube	260,911	38.6	14
Cocody	447,055	76.1	91
Koumassi	433,139	4	17
Marcory	249,858	12.6	31
Plateau	7,488	8.9	35
Port-Bouet	419,033	60.5	25
Treichville	102,580	11.4	20
Yopougon	1,071,543	117	77

Even though Abidjan is the economic capital of Côte d’Ivoire, it has the highest levels of income inequality when compared to the rest of the country. Adjame, Marcory and Treichville are the city’s poorest communes; Cocody is its wealthiest commune; Plateau is the administrative and commercial commune with skyscrapers; and Yopougon and Abobo are the most populous communes of the city, including some slum housing and communal areas [50].

In sum, the city of Abidjan has several characteristics that make it a suitable environment on which to focus our research questions. In particular, (1) it is the wealthiest and most populated city in Côte d’Ivoire; (2) it has high levels of mobile phone adoption; (3) there are relevant demographic indicators available for each of its 10 communes; and (4) the disparity in socio-economic indicators across districts is wide [50], with its slums under the shadows of its skyscrapers [56].

Next, we shall describe the socio-economic indicators that we analyze in our study, and then outline the five network metrics that are related to social capital both in the communication and the mobility networks and for each of the 10 communes in the city of Abidjan.

### Socio-economic indicators

In this section we describe in detail the socio-economic indicators we correlate with the communication and mobility network characteristics of the geographical areas under investigation. We focus not only on indicators of economic well-being, but also on proxies for civic engagement and democratic participation, and public safety and security.

#### Economic well-being

Traditionally, different metrics have been used to capture the economic development of a country, such as the Gross Domestic Product (GDP) and the Gross National Income (GNI). However, such indicators are neither well defined nor accessible at a district (commune) level, and especially for the 10 communes of the city of Abidjan. Therefore, we rely on two alternative data sets: (i) the land use master plan of Abidjan [57], and (ii) the budgets for the 10 communes from 1990 [58], as shown in Table A in S1 File. We use this data to obtain suitable indicators for economic development, and we study the association between these indicators and the social capital metrics described in the following sections.

Previous qualitative studies have suggested that: (1) Adjame, Marcory, and Treichville can be regarded as poor areas [50]; (2) Cocody is a rich, residential commune, as reflected by its large percentage of expatriates and consulates located within its boundaries [59]; (3) Abobo is a residential low-class area [59]; (4) Adjame is a commercial place with markets and a bus station [59]; and (5) Plateau is the business district.

Moreover, data on land usage provides relevant information regarding the economic status of the communes, particularly given that urban residents in some of Abidjan’s communes live in slums. We obtained land use data from the urban master plan of Abidjan published in March 2015 [57]. The plan was constructed through the collaboration between the Japan International Cooperation Agency (JICA) and the Ministry of Construction, Housing, Sanitation and Urban Development (MCLAU: http://open_jicareport.jica.go.jp/pdf/12230603_01.pdf). Data on land usage includes several categories, such as *informal settlement*, *commercial/office land*, *educational land*, and *security offices*. We use the percentage of *informal settlement* in each commune to assess its economic condition. We obtained additional data on land use from the thesis by Quonan Christian Yao-Kouassi on the spatial distribution of housing settlements in Abidjan [60]. Table 3 summarizes all variables used in our study to measure economic prosperity.

**Table 3:** Spatial distribution of settlements in Abidjan. Values are in hectares, and each percentage refers to the ratio between corresponding land usage and total commune land coverage. Source: [57] for informal settlement, and [60] for the remaining three variables.

Commune	Informal settlement	Slums	Shared houses	Unshared houses
Abobo	661	909.43	23.48	48.52
	0.09%	89.99%	2.32%	4.80%
Adjamé	86	171.08	49.49	64.11
	0.07%	55.44%	16.04%	20.78%
Attécoubé	233	251.09	5.27	49.82
	0.05%	73.01%	1.53%	14.49%
Cocody	128	94.43	143.78	846.93
	0.01%	8.15%	12.42%	73.14%
Koumassi	63	130.42	12.52	100.90
	0.05%	40.29%	3.87%	31.17%
Marcory	45	87.85	27.57	357.01
	0.03%	18.16%	5.70%	73.80%
Plateau	0.00	1.85	16.37	39.78
	0.00%	3.19%	28.22%	68.59%
Port-Bouet	1,278	28.14	20.70	153.58
	0.13%	4.79%	3.52%	26.13%
Treichville	1	65.83	23.11	45.64
	0.001%	47.87%	16.80%	33.19%
Yopougon	323	526.94	29.26	541.45
	0.03%	45.17%	2.51%	46.41%

#### Civic engagement and democratic participation

People’s democratic participation is significantly influenced by the underlying network of social interactions connecting them [61]. We use the number of voters and the percentage of voters out of all registered citizens to investigate associations between democratic participation and various types of the underlying social structures (*i.e*., bonding and bridging structures). Specifically, we use data obtained from the 2011 presidential election, as shown in Table 4. The percentage of participation varies significantly across communes, ranging from 15.7% in Cocody to 36.8% in Treichville.

**Table 4:** Participation of Abidjan’s communes in the 2011 elections.

Commune	No. voters	Participation (%)
Abobo	354,535	30.6
Adjame	102,423	35
Attecoube	82,234	30.4
Cocody	213,668	15.7
Koumassi	143,276	30.8
Marcory	83,748	20.6
Plateau	24,091	20.9
Port-Bouet	97,617	20.3
Treichville	46,986	36.8
Yopougon	450,717	19.5

#### Public safety and security

To measure public safety and security, we use the number of stolen cars from 2009 and 2010, obtained from the Statistical Department of Côte d’Ivoire [62], as shown in Table 5. In addition to these records, we also analyze the land use of security-related buildings in each commune by using data from the urban master plan of Abidjan published in March 2015 [57].

**Table 5:** Number of stolen cars in Abidjan. Source: INS Annuaire des Statistiques Démographiques et Sociales, 2009-2010.

Commune	2009	2010	Total
Abobo	82	46	128
Adjamé	81	42	123
Attécoubé	0	0	0
Cocody	198	142	340
Koumassi	0	0	0
Marcory	171	81	252
Plateau	0	0	0
Port-Bouët	97	43	140
Treichville	0	0	0
Yopougon	164	74	238

### The mobility and communication networks

From the mobile data set, we construct two different place-centric networks: (i) a *communication* network, denoted by *G*_*c*_, obtained from traffic from cell tower to cell tower available in Subset-1 in our data set; and (ii) a *mobility* network, denoted by *G*_*m*_, constructed using consecutive calls by the same user from Subset-2 in our data set. In both networks, *A* is the set of all cell towers in the country, *A* = {*a*_1_, *a*_2_, …, *a*_*n*_}, and *U* is the set of all unique users in our data set, *U* = {*u*_1_, *u*_2_, …, *u*_*n*_}.

#### The communication network

Since we do not have data on which user made or received calls, we model the information flow through the hourly aggregate records of call volumes in each initiating and receiving cell tower, which is available in Subset-1. We use the total number of phone calls that took place hourly between each pair of cell towers *a*_*i*_ and *a*_*j*_ in *A*. From this data, we construct the directed communication graph *G*_*c*_(*A*, *E*^*C*^, *W*^*C*^), where: (i) *A* = {*a*_1_, *a*_2_, …, *a*_*n*_} is the set of nodes represented by the cell towers; (ii) $E^{C}=\left\{e_{\left(a_{1},a_{2}\right)}^{C},\ldots \right\}$ is the set of directed edges $e_{\left(a_{i},a_{j}\right)}^{C}$ connecting node *a*_*i*_ to node *a*_*j*_ such that an edge $e_{\left(a_{i},a_{j}\right)}^{C}$ is established from cell *a*_*i*_ to cell *a*_*j*_ when there is at least one user who made a phone call from *a*_*i*_ to *a*_*j*_; and (iii) $W^{C}=\left\{w_{e_{\left(a_{1},a_{2}\right)}^{C}}^{C},\ldots \right\}$ is the set of weights associated with edges, where the weight of each edge $w_{e_{\left(a_{i},a_{j}\right)}^{C}}^{C}$ is equal to the total number of phone calls that took place from *a*_*i*_ to *a*_*j*_ across the whole observation period. The resulting weighted adjacency matrix is asymmetric and its order is given by the number of cell towers in the data set (*i.e*., *N*[*A*] × *N*[*A*], where *N*[*A*] is the cardinality of the set *A*).

Notice that phone calls that originated from, and were directed at, the same cell tower are disregarded. In this way, we neglect self-loops in the communication network, which could be relevant only for capturing forms of collective social capital stemming from communication within the same community. However, since our objective is to uncover the role played by bonding and bridging ego-centered social structures at the level of the cell tower, self-loops play no role in our study and therefore do not contribute to the formalization of the network. The same argument also applies to the construction of the mobility network (see below), in which self-loops do not affect the interplay between bonding and bridging structures.

#### The mobility network

The mobility network is constructed based on the locations of the cell towers to which any two consecutive calls by the same user are associated. Specifically, a movement by *u*_*i*_ is established from cell *a*_*i*_ to cell *a*_*j*_ if *u*_*i*_ makes two subsequent calls, one from cell tower *a*_*i*_ and the other from cell tower *a*_*j*_. Formally, the movement $P_{u_{i}}\left(a_{i},a_{j}\right)$ of user *u*_*i*_ from cell *a*_*i*_ to cell *a*_*j*_ is defined as:

$$\begin{array}{c} P_{u_{i}}\left(a_{i},a_{j}\right)=\{\begin{array}{cc} 1 & \text{if}\;u_{i}\;\text{makes}\;\text{a}\;\text{call}\;\text{from}\;\text{cell}\;a_{i}\;\text{and}\;\text{a}\;\text{subsequent}\;\text{call}\;\text{from}\;\text{cell}\;a_{j}\\ 0 & \text{otherwise}\; \end{array} \end{array}$$ (1)

where *a*_*i*_, *a*_*j*_ ∈ *A*, and *a*_*i*_ ≠ *a*_*j*_.

To construct the mobility graph, we aggregate such movements over the whole observation period, which in our case includes 140 days. Formally, the mobility graph can be defined as *G*_*m*_(*A*, *E*^*M*^, *W*^*M*^), where *A* = {*a*_1_, *a*_2_, …, *a*_*n*_} is the set of nodes represented by the cell towers, $E^{M}=\left\{e_{\left(a_{1},a_{2}\right)}^{M},\ldots \right\}$ is the set of directed edges $e_{\left(a_{i},a_{j}\right)}^{M}$ connecting node *a*_*i*_ to node *a*_*j*_ such that

$$\begin{array}{c} e_{\left(a_{i},a_{j}\right)}^{M}=\{\begin{array}{cc} 1 & \text{if}\;\exists u_{i}\in U.\;P_{u_{i}}\left(a_{i},a_{j}\right)=1\\ 0 & \text{otherwise}\; \end{array} \end{array}$$ (2)

and $W^{M}=\left\{w_{e_{\left(a_{1},a_{2}\right)}^{M}}^{M},\ldots \right\}$ is the set of weights associated with edges, which reflects the number of movements users made between the connected nodes. In particular, we compute the weight of each edge by using all users’ movements between the connected cell towers:

$$\begin{array}{c} w_{e_{\left(a_{i},a_{j}\right)}^{M}}^{M}=\sum_{u_{i}\in U}P_{u_{i}}\left(a_{i},a_{j}\right). \end{array}$$ (3)

As with the communication network, the resulting weighted adjacency matrix is asymmetric, with an order equal to *N*[*A*] × *N*[*A*].

We construct the mobility network using the data in Subset-2 with the geographic granularity given by the locations of cell towers. In this network, information is assumed to flow between connected pairs of nodes, and therefore between the locations with which the nodes are associated. Thus, as people move from one location to the next, they act as vehicles of information across space.

## Network metrics for closed and open structures

In this section we describe the five network metrics that we compute to uncover social capital. These five metrics are: (i) degree centrality, (ii) betweenness centrality, (iii) effective size, (iv) efficiency, and (v) the local clustering coefficient [30, 63, 64]. Notice that both the communication (*G*_*c*_) and the mobility networks (*G*_*m*_) are directed and weighted graphs. However, not all of our metrics leverage these characteristics. Indeed, some of our metrics (*i.e*., effective size and efficiency) use an undirected graph and one of our metrics (*i.e*., the clustering coefficient) assumes an undirected and unweighted graph. In these cases, we transform the directed graph into an undirected one by computing the average weight of each edge.

We begin by introducing the simplest centrality measure (degree centrality) that we use to detect the value a node (*i.e.,* a cell tower) can extract solely from the direct connections to its local neighbors. Next we broaden our perspective to also account for connections between the nodes’ first neighbors or even for connections beyond first neighbors and local networks. To this end, we group the network metrics into two groups, depending on which type of social capital they are aimed to capture [24, 65].

The metrics included in the first group –betweenness centrality, effective size, and efficiency– share the idea that a node can draw influence, power or importance from the absence of edges in the network [4, 10]. In this sense, they capture the degree to which nodes span structural cleavages, holes or discontinuities in the network, and can therefore act as potential intermediaries for others to interact and communicate with one another. This is indeed the idea of social capital as the *bridging potential* that nodes can tap from open structures rich in brokerage opportunities. Conversely, the metric in the second group –the local clustering coefficient– is based on the idea that nodes can draw value from the presence of edges in their local network [1]. Specifically, it captures the degree to which a node’s contacts are also connected with each other, and therefore the triads centered on the node are closed into triangles. This metric can thus be used to test hypotheses on social capital rooted in the value of social cohesion and in the *bonding* potential that nodes can tap from closed structures rich in third-party relationships.

### Degree centrality

In graph theory, the degree centrality of a node refers to the number of connections incident upon the node [66, 67]. In the context of the communication network, a connection is established whenever a call takes place from one cell tower to another. In the mobility network, a connection between any two locations is established whenever the same mobile user has made consecutive phone calls connected to the cell towers located in these two locations. In our current work, a connection was established between locations if either an inbound or an outbound link between the corresponding cell towers was created. Reciprocity is thus not accounted for. It has been widely suggested that degree centrality quantifies the potential amount of information a node may have access to [66]. Thus, centrality can be used to measure the amount of resources an individual or, as in our case, a place can mobilize through the direct contacts. In this sense it can be seen as a measure of the value that can be extracted from one’s local network. From this perspective, for instance, in Borgatti *et al*. [63] degree centrality has been positively related with social capital. However, degree in itself is not sufficient for capturing the potential benefit a node can derive from the network. Indeed, degree alone does not reflect the value a node can extract beyond the direct connections, nor the bridging or bonding potential of the node’s ego-centered network. It is therefore necessary to define additional metrics to characterize the bridging and bonding social capital.

### Network metrics for open bridging structures

This section will outline three network metrics that aim to capture brokerage opportunities and thus the value that actors can extract from the bridging potential of the underlying network (bridging social capital). The first of these metrics –betweenness centrality– is a global centrality measure that accounts for edges lying across the whole network. The other two measures –effective size and efficiency– are local measures that only reflect the topology of the node’s ego-centered network, that is the node’s direct connections to its first neighbors and the connections between these neighbors.

#### Betweenness centrality

Betweenness centrality measures the extent to which a node lies on the paths that connect other nodes [66–68]. Nodes with large betweenness centrality may gain considerable influence within a network by virtue of their control over information flowing to and from other nodes. These nodes are also the ones whose removal from the network will most disrupt communication between other nodes because they lie on the largest number of paths along which messages travel [63]. From this perspective, it can be argued that betweenness centrality quantifies the bridging potential of a node that leverages on the lack of edges connecting other nodes. Thus, betweenness centrality can be used to test hypotheses on the value that can be extracted from open social structures providing nodes with brokerage and bridging opportunities.

To measure betweenness centrality, we transform our directed weighted graph into an undirected weighted one. The transformation is carried out by taking the maximum weight of either directed link. In so doing, the underlying assumption is that information flows in either direction when there is at least one directed link from one to another. Finally, to compute weighted betweenness centrality on such undirected graph, we use the *MATLAB* betweenness centrality function.

#### Effective size

The effective size of node *a*_*i*_’s ego-centered network aims to capture the degree to which the node’s first neighbors are redundant. Formally, it can be expressed as a function of the following two matrices: the transition matrix **T**, and the marginal strength matrix **M**. The element $t_{a_{i},a_{l}}$ of matrix **T** measures the proportion of *a*_*i*_’s network time and energy invested in the relationship with node *a*_*l*_, as defined in [4, 24]:

$$\begin{array}{c} t_{a_{i},a_{l}}=\frac{w_{a_{i},a_{l}}+w_{a_{l},a_{i}}}{\sum_{a_{m}}\left(w_{a_{i},a_{m}}+w_{a_{m},a_{i}}\right)}, \end{array}$$ (4)

where $w_{a_{i},a_{l}}+w_{a_{l},a_{i}}$ is the sum of the weights of the two links connecting *a*_*i*_ to *a*_*l*_, while $\sum_{a_{m}}\left(w_{a_{i},a_{m}}+w_{a_{m},a_{i}}\right)$ is the total strength of node *a*_*i*_. This is the sum of node *a*_*i*_’s out-strength, $s_{a_{i}}^{out}=\sum_{a_{m}}w_{a_{i},a_{m}}$, and in-strength, $s_{a_{i}}^{in}=\sum_{a_{m}}w_{a_{m},a_{i}}$, *i.e*., the sum of the weights of all the incoming and outgoing links incident upon node *a*_*i*_. Notice that by definition $0\leq t_{a_{i},a_{l}}\leq 1$ for all *a*_*i*_, *a*_*l*_ with $t_{a_{i},a_{l}}=0$ if there is neither a link from node *a*_*i*_ to node *a*_*l*_, nor a link from node *a*_*l*_ to node *a*_*i*_. The transition matrix **T** is row-stochastic: $\sum_{a_{j}}T_{a_{i},a_{l}}=1$.

The entry $m_{a_{j},a_{l}}$ of the marginal strength matrix **M** is defined as:

$$\begin{array}{c} m_{a_{j},a_{l}}=\frac{w_{a_{j},a_{l}}+w_{a_{l},a_{j}}}{{\text{max}}_{a_{m}}\left(w_{a_{j},a_{m}}+w_{a_{m},a_{j}}\right)}. \end{array}$$ (5)

Notice that $0\leq m_{a_{j},a_{l}}\leq 1$ for all *a*_*j*_, *a*_*l*_ with $m_{a_{j},a_{l}}=0$ if there is neither a link from node *a*_*j*_ to node *a*_*l*_ nor from *a*_*l*_ to *a*_*j*_. Finally, the two matrices **T** and **M** defined above are asymmetric.

Thus, the effective size $S_{a_{i}}$ of node *a*_*i*_’s ego-centered network can be formalized as follows:

$$\begin{array}{c} S_{a_{i}}=\sum_{a_{j}\in N_{a_{i}}}[1-\sum_{a_{l}}t_{a_{i},a_{l}}m_{a_{j},a_{l}}], \end{array}$$ (6)

where $N_{a_{i}}$ is the set of node *a*_*i*_’s neighbors and *a*_*l*_ ≠ *a*_*i*_, *a*_*j*_.

Effective size was originally introduced by Burt [25] to quantify the degree to which node *a*_*i*_’s ego-centered network is rich in structural holes, and thus provides the node with bridging potential and brokerage opportunities.

#### Efficiency

Efficiency can be defined as the normalized value of effective size, namely the ratio between node *a*_*i*_’s effective size and degree. Formally,

$$\begin{array}{c} {\text{Efficiency}}_{a_{i}}=\frac{S_{a_{i}}}{{\text{k}}_{a_{i}}}, \end{array}$$ (7)

where $k_{a_{i}}$ is the degree of node *a*_*i*_. Like effective size, efficiency quantifies the bridging potential of a node. As it is normalized by the degree, the measure takes value within 0 and 1. The closer it is to 1, the greater the bridging potential of *a*_*i*_’s ego-centered network. That is, a unitary value means that each of node *a*_*i*_’s neighbors is non-redundant, whereas a value close to *zero* would imply that all of *a*_*i*_’s first neighbors are redundant, and are thus unable to provide unique and non-overlapping information or resources.

### Network metric for closed bonding structures

In this section, we describe the metric we used for capturing the social capital that originates from closed bonding structures, namely the local clustering coefficient. This is a node-level local measure of social cohesion that captures how closed the node’s ego-centered network is.

#### The local clustering coefficient

In order to capture the social cohesion of node *a*_*i*_’s ego-centered network, we now focus on *a*_*i*_’s local clustering coefficient, ($C_{a_{i}}$), which measures the probability that node *a*_*i*_’s first neighbors are also connected with each other [67, 69]. Formally, the local clustering coefficient can be formalized as

$$\begin{array}{c} C_{i}=\{\begin{array}{cc} \frac{E[G_{a_{i}}]}{\frac{k_{a_{i}}\left(k_{a_{i}}-1\right)}{2}} & \text{for}\;k_{a_{i}}\geq 2\\ 0 & \text{for}\;k_{a_{i}}=0,1 \end{array} \end{array}$$ (8)

where $E[G_{a_{i}}]$ is the number of edges included in the subgraph $G_{a_{i}}$ induced by the set $N_{a_{i}}$ of node *a*_*i*_’s first neighbors, (*i.e*., $E[G_{a_{i}}]$ is the number of edges linking *a*_*i*_’s neighbors), and $k_{a_{i}}$ is the degree of node *a*_*i*_. Notice that $C_{a_{i}}$ is normalized such that it varies between 0 and 1, $0\leq C_{a_{i}}\leq 1$. In particular, it takes the value of 0 when none of *a*_*i*_’s neighbors are connected with each other, and takes the value of 1 when all pairs of *a*_*i*_’s neighbors are connected. The local clustering coefficient captures the degree to which the triads centered on *a*_*i*_ are closed into triangles, and thus can suitably measure the degree to which node *a*_*i*_’s ego-centered network is closed and rich in third-party relationships. In this sense, the local clustering coefficient has been widely used in studies of social capital to investigate the benefits of closed structures [24].

Furthermore, the local clustering coefficient is formally related to Burt’s redundancy measure. To formalize this relationship, we begin by showing how effective size can be expressed as the difference between the number of node *a*_*i*_’s first neighbors and *a*_*i*_’s ego-centered network redundancy. Formally,

$$\begin{array}{c} S_{a_{i}}=N\left[G_{a_{i}}\right]-\frac{2E[G_{a_{i}}]}{N[G_{a_{i}}]}, \end{array}$$ (9)

where $N[G_{a_{i}}]$ is the number of nodes included in the subgraph $G_{a_{i}}$ (*i.e*., the cardinality of the set $N_{a_{i}}$), $\frac{2E[G_{a_{i}}]}{N[G_{a_{i}}]}$ is the redundancy of node *a*_*i*_’s ego-centered network, measured as the average degree of *a*_*i*_’s neighbors. In turn, $\frac{2E[G_{a_{i}}]}{N[G_{a_{i}}]}$ is simply the ego-centered network density, scaled by a factor of $N[G_{a_{i}}]-1$.

Moreover, it has been demonstrated that effective size is formally related to the local clustering coefficient as follows [24]:

$$\begin{array}{c} S_{a_{i}}=k_{a_{i}}-\left(k_{a_{i}}-1\right)C_{a_{i}}. \end{array}$$ (10)

where $k_{a_{i}}=N\left[G_{a_{i}}\right]$. If we now plug Eq 9 into Eq 10, we obtain:

$$\begin{array}{c} C_{a_{i}}=\frac{\frac{2E[G_{a_{i}}]}{N[G_{a_{i}}]}}{k_{a_{i}}-1}=\frac{{\text{Redundancy}}_{a_{i}}}{k_{a_{i}}-1} \end{array}$$ (11)

Thus, the local clustering coefficient can be expressed in terms of local network redundancy. The more redundant a node’s contacts are, the larger the proportion of triads centered on the node that close into triangles, and thus the higher the local clustering coefficient. Note that local clustering and effective size vary with redundancy in opposing ways (see Eqs 9 and 11). In this sense, it has been argued that the former can be seen as a mirror image of the latter [24]. Indeed, while effective size is sensitive to the *absence* of edges between node *a*_*i*_’s alters, and thus captures the non-redundancy of edges in *a*_*i*_’s ego-centered network and its bridging potential, the local clustering coefficient is sensitive to the *presence* of edges in *a*_*i*_’s ego-centered network, and therefore captures the redundancy of edges in the network as well as its social cohesion and bonding potential. In this sense, the two measures, and thus the two local network topologies they aim to quantify, can be regarded as the two faces of the same coin [24].

#### Temporal and spatial aggregation

We perform temporal and spatial aggregation as follows. We first temporally aggregate the data for the entire observation period (*i.e*., 140 days) and for each network (*i.e*., the communication and mobility networks). Next, we compute the five metrics for each cell tower and then we aggregated them spatially at the commune level, because the demographic and socio-economic indicators publicly available are only provided at this level of granularity. Notice that the number of cell towers in each commune is different, as shown in Table 2. In particular, we compute the mean, the standard deviation, the median, the mode, the minimum, and the maximum of each distribution for each metric. In Tables 6 and 7 we report the mean values of our five metrics for both communication and mobility networks, respectively. It is worth noting that the network metrics are computed using data covering the cell towers of the whole country (*i.e*., 1, 238 cell towers) and not data restricted to the cell towers located within the Abidjan boundaries (*i.e*., 378 cell towers). For example, for computing the communication network degree centrality of Abobo, one of the communes of Abidjan, we consider the call connections between each cell tower in Abobo and the cell towers located anywhere in the country, within and beyond Abidjan. Summaries of the other descriptive statistics are presented in Tables B—K in S1 File.

**Table 6:** Mean values of the degree centrality, betweenness centrality, effective size, efficiency, and local clustering coefficient in the communication network. These measures were calculated for each of the ten communes of Abidjan.

		Open structure			Closed structure
Commune	Degree	Between. centrality	Eff. size	Efficiency	Local clust. coeff.
Abobo	1200.200	1128.866	624.555	0.521	0.946
Adjame	1195.565	1123.036	609.005	0.509	0.946
Attecoube	1202.538	1147.459	619.986	0.516	0.945
Cocody	1141.789	1054.691	560.872	0.476	0.948
Koumassi	1188.118	1125.058	654.206	0.551	0.947
Marcory	1025.103	841.657	573.949	0.552	0.958
Plateau	1001.588	789.809	427.779	0.394	0.961
Port-Bouet	1067.750	896.124	607.666	0.568	0.956
Treichville	1113.850	950.254	612.798	0.550	0.954
Yopougon	1175.697	1143.794	599.460	0.502	0.946

**Table 7:** Mean values of the degree centrality, betweenness centrality, effective size, efficiency, and local clustering coefficient in the mobility network. These measures were calculated for each of the ten communes of Abidjan.

		Open structure			Closed structure
Commune	Degree	Between. centrality	Eff. size	Efficiency	Local clust. coeff.
Abobo	417.489	2642.071	369.086	0.881	0.666
Adjame	533.174	7693.997	474.576	0.884	0.555
Attecoube	466.308	4076.686	412.843	0.882	0.626
Cocody	370.258	904.283	319.693	0.850	0.756
Koumassi	406.353	1431.543	366.306	0.901	0.690
Marcory	285.690	497.009	256.203	0.885	0.805
Plateau	324.176	845.653	276.827	0.811	0.776
Port-Bouet	276.000	861.109	253.292	0.916	0.772
Treichville	369.250	1067.158	331.506	0.894	0.737
Yopougon	394.880	2021.052	350.846	0.881	0.688

## Results

In this section we present the results obtained by computing bivariate correlations between each of the five social capital network metrics and a number of district-level socio-economic indicators related to (i) economic well-being, (ii) civic engagement and democratic participation, and (iii) public safety and security. These results are obtained by computing Pearson’s *r*, Spearman’s *ρ*, and Kendall’s *τ* correlation coefficients. For each district, we computed the mean, the standard deviation, the median, the mode, the minimum and the maximum of each node’s network metrics in order to have single values for each district to correlate with the corresponding socio-economic indicators. While by aggregating values we inevitably lose high-granularity information, we can still detect different behaviors between poor and wealthy regions. In the following subsections, we shall report the results obtained using the mean values of our network metrics. We also estimated linear ordinary-least squares and negative binomials models of districts’ economic well-being, civic engagement and democratic participation, and public safety and security. To avoid problems arising from multi-collinearity, only two distinct subsets of variables at a time could be used as covariates in each model. Estimated parameters are reported in Tables L—O in S1 File. Results are consistent with the correlation coefficients reported below.

### Economic well-being

**Fig 2:**
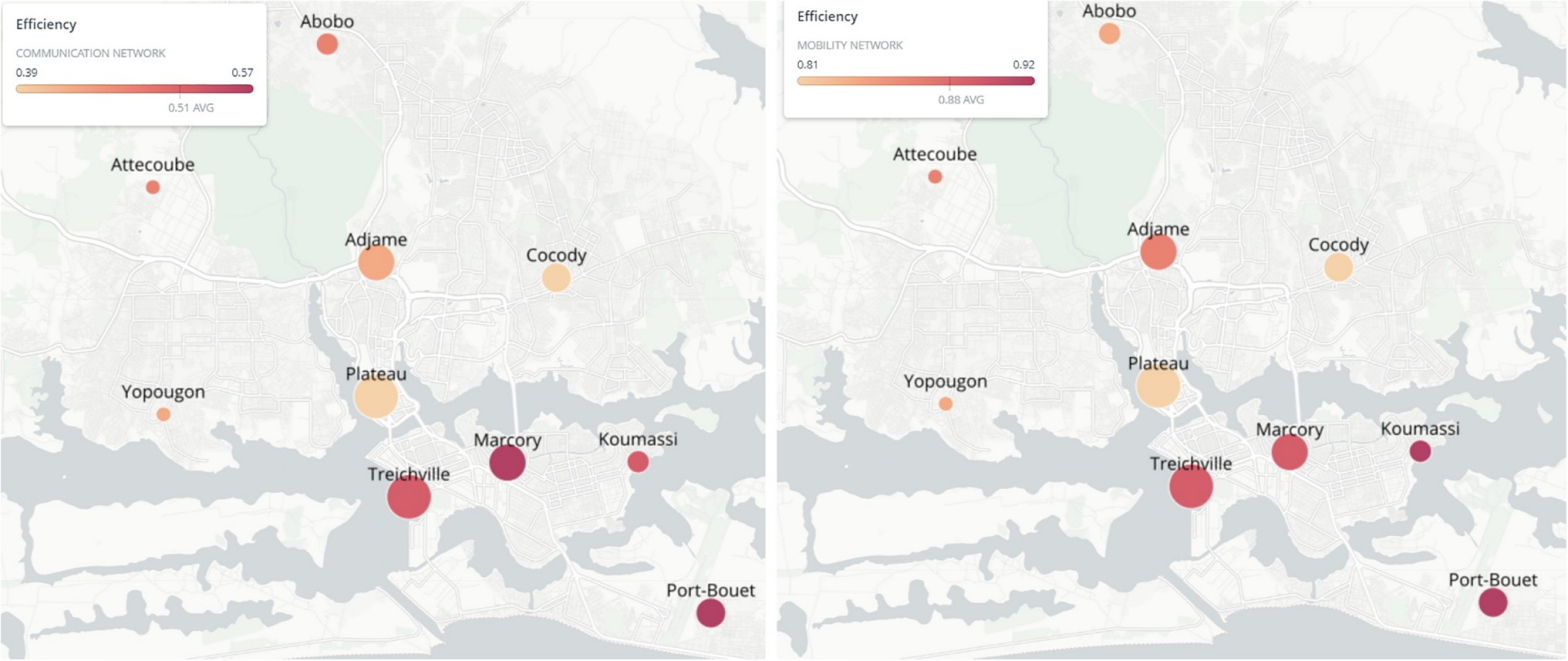
Association between efficiency and revenue budget per person in the communication (left-hand panel) and mobility (right-hand panel) networks. In both networks, the nodes refer to the communes in Abidjan. The color of each node is proportional to its corresponding efficiency, while its size is proportional to the revenue budget per person in the corresponding commune.

Interestingly, we find negative correlations between measures for open bridging structures (*i.e*., effective size and efficiency) and indicators of economic wealth, while positive correlations are found between the same measures and indicators of economic deprivation. For example, the effective size based on the communication network is negatively associated with the revenue budget per person (Pearson’s *r* = −0.91, *p* = 0.00021) and the capital budget per person (Pearson’s *r* = −0.91, *p* = 0.00027). Moreover, the communication network-based efficiency is negatively associated with the revenue budget per person (Pearson’s *r* = −0.81, *p* = 0.00468) and with the capital budget per person (Pearson’s *r* = −0.81, *p* = 0.0042), and the mobility network-based efficiency is negatively associated with the revenue budget per person (Pearson’s *r* = −0.80, *p* = 0.0057) and with the capital budget per person (Pearson’s *r* = −0.81, *p* = 0.00408). Thus, our findings suggest that, both in the communication and mobility networks, economic well-being is positively associated with closed and cohesive social structures, poor in brokerage opportunities and rich in third-party relationships. Table 8 summarizes the statistically significant results (*p* < 0.05). For illustrative purposes, Fig 2 shows the association between efficiency and revenue budget per person of communes in the communication and mobility networks.

**Table 8:** Summary of results on economic well-being indicators based on both communication and mobility networks. Only statistically significant correlations are reported (*p* < 0.05). Note: ‘*’: *p* < 0.05; ‘**’: *p* < 0.01; ‘***’: *p* < 0.001.

	Revenue budget per person		Capital budget per person		Percentage of land covered by unshared houses		Percentage of land covered by shared houses		Percentage of land covered by slums		Informal settlement land use	
Degree	Comm. -0.64*	Mob.	Comm.	Mob.	Comm. -0.68*	Mob.	Comm.	Mob.	Comm. 0.78**	Mob. 0.72**	Comm.	Mob.
**Open structures:**												
Betweenness												
centrality	-0.63*								0.73*			
Effective size	-0.91***		-0.91***		-0.66*		-0.76**			0.73**		
Efficiency	-0.81**	-0.80**	-0.81**	-0.81**			-0.69*	-0.71*				0.63*
**Closed structures:**												
Local clust. coeff.	0.64*		0.62*			0.7*			-0.72*	-0.75**		

### Civic engagement and democratic participation

Putnam has documented associations between high levels of democratic participation and civic engagement and dense networks, which typically correspond to high level of social cohesion and few structural holes [2, 70]. Instead, our findings show an opposite trend. In particular, we find a positive correlation between effective size and democratic participation, both in the communication (Pearson’s *r* = 0.66, *p* = 0.00021) and the mobility (Spearman’s *ρ* = 0.66, *p* = 0.0375) networks. Thus and according to our data, bridging open network structures, rich in brokerage opportunities, are positively associated with the individuals’ tendency to vote. Table 9 summarizes the statistically significant results (*p* < 0.05).

**Table 9:** Summary of results for civic engagement and democratic participation, based on both the communication and mobility networks. Only statistically significant correlations are reported (*p* < 0.05). Note: ‘*’: *p* < 0.05; ‘**’: *p* < 0.01; ‘***’: *p* < 0.001. By default Pearson’s correlation coefficients are shown. (S) stands for Spearman’s *ρ*.

Democratic participation			
		Communication	Mobility
**Open structures:**	Effective size	0.66* (S)	0.66*
**Closed structures:**	Local clustering coefficient		-0.64*

### Public safety and security

Our findings show that districts that are more connected (*i.e*., with higher degree), and are more diverse, with more bridging opportunities (*i.e*., with high betweenness centrality and effective size), also have lower numbers of police stations and other public safety and security offices. Degree is negatively associated with the proportion of land covered by security offices (Pearson’s *r* = −0.71, *p* = 0.02127). Similarly, effective size and betweenness centrality are inversely correlated with the proportion of land covered by security offices (Pearson’s *r* = −0.60, *p* = 0.06505 and Pearson’s *r* = −0.68, *p* = 0.0318, respectively). Conversely, districts showing higher social cohesion (*i.e*., high levels of local clustering coefficient) have a larger number of security offices than districts with lower cohesion (Pearson’s *r* = 0.63, *p* = 0.050). These results are consistent across both the communication and mobility networks. Table 10 summarizes the statistically significant results (*p* < 0.05).

**Table 10:** Summary of results for public safety and security based on both the communication and mobility networks. Only statistically significant correlations are reported (*p* < 0.05). Note: ‘*’: *p* < 0.05; ‘**’: *p* < 0.01; ‘***’: *p* < 0.001. By default Pearson correlation coefficients are shown. (K) stands for Kendall *τ*.

Security				
	Security (land coverage)		Stolen cars (2009)	
	Comm.	Mob.	Comm.	Mob.
Degree	-0.71*	-0.63*		
**Open structures:**				
Betweenness centrality	-0.67*			
Effective Size	-0.60*	-0.66*	-0.50* (K)	
**Closed structures:**				
Local clust. coeff.	0.63*	0.70*		

Interestingly, our results also show that bridging districts (*i.e*., with high effective size) experience lower numbers of stolen cars (Kendall’s *τ* = −0.50, *p* = 0.0436314). However, we find a statistically significant result only for the value computed on the communication network. Finally, we also find a negative association between (the modal value of) efficiency (measuring the extent to which a node’s social ties provide bridging opportunities) and the number of stolen cars in 2009 (Pearson’s *r* = −0.63, *p* = 0.0.05093) and in 2010 (Pearson’s *r* = −0.72, *p* = 0.0199) for mobility networks. Notice that, for the sake of clarity and consistency, Table 10 does not report correlation coefficients for efficiency. Indeed the Pearson’s correlation coefficients in 2009 and 2010 are statistically significant only for the mode of efficiency, whereas all Tables report correlations between average values. The average value of efficiency is statistically significantly correlated with number of stolen cars only in 2009.

## Discussion

Measuring social capital is typically a challenging task, as both social interactions and the value that such interactions bring to individuals, groups, or places are hard to model and quantify. In general, there is a lack of ground truth data about the benefits that can be derived from social interactions. In particular, the problem of uncovering the structural foundations of social capital becomes even harder when the population under study is sampled from developing economies, for which there is paucity of high-quality, high-granularity socio-economic data.

In this study, we took a step in this direction and proposed to use aggregated mobile phone usage data and socio-economic indicators to overcome these difficulties in the measurement and detection of social capital in a developing economy. In particular, drawing on mobile phone usage data, we constructed the place-centric communication and mobility networks and used publicly available socio-economic indicators for the 10 districts in the city of Abidjan. Our findings shed a novel light on the relationship between bridging and bonding social structures of geographical places and the role they have in eliciting social capital in a developing country.

### Economic well-being

We found a negative correlation between bridging structures and wealth indicators (*e.g*., wealthy residential areas) both in the communication and mobility networks, and a positive correlation between bridging structures and poverty indicators. Thus, our results do not seem to corroborate the findings of Eagle *et al*. that the diversity of individuals’ social relationships is strongly correlated with the economic development of their communities [49]. In particular, their results suggested that socio-economic opportunities in a given community in the United Kingdom increase with the number of structural holes in the ego-centered networks of the members. Notice that, unlike the work by Eagle *et al*. [49] but in qualitative agreement with the studies by Smith-Clarke *et al*. [51] and Mao *et al*. [50], in our analysis the cell towers, and not the individuals, are the nodes of the mobility and communication networks, which are then aggregated at the level of the commune. Underpinning our approach is the assumption that the structural position of a cell tower and the corresponding commune in the overall network can serve as source of social capital to the community of people that for various reasons happen to be present within the areas (*i.e*., the resident population, the daily commuters, workers, students, visitors and passers-by). A direct comparison of our results with the ones obtained by Eagle *et al*. [49] should therefore be taken with caution, and future research should further explore these contrasting outcomes by analyzing and comparing data from developed and developing economies.

Our results are in agreement with the studies conducted by Coleman on the role that *community closure* plays in nurturing economic well-being [1]. In particular, Coleman used the term ‘closure’ to refer to a tightly knit social structure, rich in third-party social relationships, in which community members can support one another, cooperate, enforce social norms, and sanction free-riding and deviant behaviors. Another comparison can also be made between our study and a recent extensive survey that Lotero *et al*. conducted among 56, 513 citizens of Medellin, Colombia, about their mobility behaviors and their socio-economic characteristics, such as age, gender, and job [71]. While once again the focus of Lotero *et al*.’s study was on individuals and not on communities or places as in our case, interestingly in qualitative agreement with our work they found that the mobility routines and travels of rich people are highly redundant and localized, while the opposite trend characterizes the mobility routines of poorer citizens [71]. This means that, in the context of a developing economy, the social interactions and mobility routines of economically disadvantaged people tend to be more diverse than the ones of richer people.

### Civic engagement and democratic participation

According to Putnam’s theories [2, 7, 30], closed and socially cohesive communities, namely communities with high levels of bonding social relationships, are expected to exhibit a higher democratic participation and civic engagement. However, in our analyses we found evidence of an opposite tendency: the denser the communities, the lower their democratic participation. In addition, our results suggest that communities with high values of bridging social structures have higher levels of democratic participation than communities with bonding structures, a finding in line with previous work in the social science literature [61, 72, 73]. Indeed, Magee [72] found a statistically significant correlation between diversity and voluntary participation and neighborhood social support, and Tindall and Cormier [73] found that network diversity, and therefore structural cleavages, predicted participation in voluntary organizations as well as political engagement.

Interestingly, the areas with higher levels of democratic participation tend to be the economically deprived districts of Abidjan. One potential explanation for this finding is that poor communities may have a larger incentive to participate in the democratic process as they are driven by the aspiration to promote changes that would lead to improvements in quality of life [74]. However, simply based on our study and correlational analysis, we are unable to conclude whether gaining access to different sources of information (*i.e*., forging bridging relationships), or people’s (low) socio-economic status or some other latent variable(s) or some combination of these is what motivates civic involvement.

### Public safety and security

Our results provide evidence that bridging social structures are associated with lower number of stolen cars than bonding structures. Thus, our findings support Jacobs’ theory of *natural surveillance*, suggesting that areas with a wide variety of functions and high diversity of people—*e.g*., gender and age diversity– are safer and have a lower number of crimes than more homogeneous areas [14, 75]. Similar results were found recently by several crime prediction data-driven studies [76–78]. Indeed in open structures, rich in brokerage opportunities, social connections between individuals tend to be weak [10] and are likely to link people with different ideas, interests and perspectives [4, 24]. Thus, open and bridging structures tend to be associated with more diversity than closed and bonding ones. In this sense, our results on the association between bridging and safety are consistent with Jacobs’ study on diversity [75].

Another interesting finding is that security offices are more likely to be built in places and communities characterized by bonding social structures. Interestingly, and according to Jacobs’ theories, such places and communities would have low levels of *natural surveillance* and therefore would need additional, external support to increase their security [75].

Our results are consistent across the communication and mobility networks. That is, the estimated associations between efficiency and economic well-being indicators, between effective size and civic engagement and democratic participation, between degree centrality, effective size, and local clustering coefficient on the one hand and public safety and security on the other are consistent across the two networks (see also Fig 2). However, the communication and mobility networks also capture distinct aspects of a place. Table 8 shows that the statistically significantly high correlations between effective size and economic well-being in the communication network have not been found in the mobility network too. This finding may be explained by contrasting Tables 6 and 7 in which the mean values of our five metrics (*i.e*., degree centrality, betweenness centrality, effective size, efficiency, and local clustering coefficient) are reported for the communication and mobility networks, respectively. Indeed the two tables show that there are communes with high values of betweenness centrality in the communication network (*e.g*., Yopougon), which do not have such high centrality in the mobility network. Moreover, the differences in betweenness centrality in the mobility network between communes are much more pronounced than in the communication network. Adjame is the commune with the highest value (significantly higher than the other communes) of betweenness centrality in the mobility network while this is not the case in the communication network. This may be attributable to the presence in Adjame of several locations that could act as commercial (*e.g*., a market) and mobility (*e.g*., an important bus station) hubs. Koumassi has the highest value of effective size in the communication network but not in the mobility network, where higher positions in the ranking by effective size are secured by other communes, such as Adjame and Attecoube.

Notice that our findings only highlight associations between variables, and not causal relationships. Thus, simply based on our study we are unable to draw conclusions on the actual structural causes that can explain high levels of socio-economic well-being or safety. This would of course require additional data and analysis which we leave for future work.

## Conclusion

In this study, we have drawn on large-scale aggregated mobile phone usage data to investigate the interplay between bridging and bonding structures of place-centric networks in eliciting social capital in Abidjan, the biggest city of Côte d’Ivoire. From the mobile data, we constructed two networks—the communication and mobility networks—that result from the communication and mobility flows taking place in the city. Next, we investigated the relationship between interaction and mobility patterns and a variety of socio-economic indicators. While some of our findings support previous empirical work [61, 76] on social capital, other findings diverge from what has been suggested by the literature [2]. For example, bridging structure, rich in brokerage opportunities, have primarily been described as wellspring of economic wealth and prosperity. By contrast, we uncovered an opposite trend: richer districts in Abidjan were found to thrive in closed bonding social structures, while poorer districts are positively associated with open bridging structures. Indeed most previous empirical studies of social capital have traditionally been focused on developed countries. Our work thus fills an important gap in the literature by shedding a new light on the structural foundations of social capital in a developing country, thus paving the way towards a more informed comparative analysis of the role of social structures in developed versus developing countries.

Our study is not without limitations. Since we only analyzed one city in a developing economy, we believe that further research is needed to better assess the degree to which our findings can be generalized to other empirical settings. Moreover, less aggregated mobile phone usage data as well as socio-economic indicators will enable further research to uncover structural sources of social capital at the level of individuals or groups, thus integrating what we have investigated in our study only at the higher level of districts and associated cell towers. For example, it would be interesting to build distinct mobility networks (or a multiplex mobility network) by considering separately users “on the move” vs “residents” vs “people who work” in the different communes. However, such an analysis would require more fine-grained temporal and spatial data, such as GPS data or data captured by probes installed in the mobile network. Our cross-sectional analysis can be further integrated and strengthened through a longitudinal study of variations over time of social capital, with a view to uncovering how opportunities emerge and disappear as a developing country’s underlying structure evolves. Finally, our work can be extended to investigate the relation and interplay over time between communication (*i.e*., the communication network) and mobility (*i.e*., the mobility network) [79].

## Supporting information

S1 File Economic indicators and network metrics. Budgets of the 10 districts (communes) of Abidjan and descriptive statistics of both communication and mobility networks.
(PDF)

S2 File Description of the data set. (TXT)

S1 Data Set Regression variables. Dependent and independent variables for 10 communes.
(CSV)

S2 Data Set Communication network data set. Abidjan’s communication network aggregated at the commune level.
(CSV)

S3 Data Set Mobility network data set. Abidjan’s mobility network aggregated at the commune level.
(CSV)
