# Supplementary material for: The bridging and bonding structures of place-centric networks: Evidence from a developing country
Source: PLoS One. 2019 Sep 5;14(9):e0221148. doi: 10.1371/journal.pone.0221148 (PMC6728049; doi:10.1371/journal.pone.0221148)
Supplement: S1 File — Budgets of the 10 districts (communes) of Abidjan and descriptive statistics of both communication and mobility networks. (PDF) [file pone.0221148.s001.pdf]

The bridging and bonding structures of place-centric networks: Evidence from a developing country

Didem Gündoğdu[1,2*], Pietro Panzarasa[3], Nuria Oliver[4], Bruno Lepri[2]

1 Department of Information Engineering and Computer Science, University of Trento, Trento, Italy
2 Center for Information Technology, Fondazione Bruno Kessler, Trento, Italy
3 Queen Mary University of London, London, United Kingdom
4 Data-Pop Alliance, New York, United States

* didemgundogdu@gmail.com

Table A. Budgets of the 10 district (communes) of Abidjan in 1990. Source: Saint-Vil, J., Tableau de bord communal, Abidjan, DCGTx, 1991.

Commune	Budget, 1990 (CFA '000)	Revenue budget (CFA francs/person)	Capital budget (CFA francs/person)
Abobo	734,524	1,405	4,262
Adjame	1,475,381	7,387	1,607
Attecoube	230,415	1,148	260
Cocody	969,471	5,038	2,494
Koumassi	731,787	2,420	744
Marcory	1,495,000	8,147	2,085
Plateau	2,213,876	164,200	25,633
Port-Bouet	1,496,000	7,099	1,767
Treichville	2,163,537	16,026	3,635
Yopougon	711,844	1,298	602

Table B. Degree centrality of communication network. Descriptive statistics of *degree centrality* based on communication network.

	Degree centrality					
**Commune**	**Mean**	**STD**	**Median**	**Mode**	**Min**	**Max**
Abobo	1,200.20	11.08	1,204	1,205	1,145	1,205
Adjame	1,195.56	26.89	1,202	1,202	1,081	1,202
Attecoube	1,202.54	3.78	1,203	1,203	1,194	1,203
Cocody	1,141.79	206.99	1,198.5	1,206	1	1,206
Koumassi	1,188.12	24.65	1,199	1,199	1109	1,199
Marcory	1,025.10	262.43	1,109	1,171	40	1,171
Plateau	1,001.59	284.52	1,184	1,196	200	1,196
Port-Bouet	1,067.7500	193.8588	1,146	1,146	376	1,146
Treichville	1,113.85	131.09	1,145.5	1,156	610	1,156
Yopougon	1,175.70	147.79	1,206	1,206	7	1,206

Table C. Descriptive statistics. Degree centrality based on the mobility network.

	Degree centrality					
**Commune**	**Mean**	**STD**	**Median**	**Mode**	**Min**	**Max**
Abobo	417.4889	77.5115	420	318	224	318
Adjame	533.1739	112.6534	497	471	271	471
Attecoube	466.3077	98.2356	455	340	340	340
Cocody	370.2584	96.8092	387	448	4	448
Koumassi	406.3529	79.4827	438	477	266	477
Marcory	285.6897	112.8827	274	371	5	371
Plateau	324.1765	170.7148	420	447	12	447
Port-Bouet	276	114.3112	265	36	36	36
Treichville	369.2500	111.0386	357	348	85	348
Yopougon	394.8800	106.6520	413	331	57	331

Table D. Descriptive statistics. Betweenness centrality based on the communication network.

	Betweenness centrality					
**Commune**	**Mean**	**STD**	**Median**	**Mode**	**Min**	**Max**
Abobo	1128.866	31.203	1133.893	987.517	987.517	1189.227
Adjame	1123.036	68.566	1134.741	851.771	851.771	1195.990
Attecoube	1147.459	68.040	1130.950	1104.335	1104.335	1370.012
Cocody	1054.691	227.319	1122.525	0.541	0.541	1175.043
Koumassi	1125.058	63.911	1139.323	911.252	911.252	1196.076
Marcory	841.657	333.818	959.499	0.074	0.074	1208.695
Plateau	789.809	427.570	1075.218	1.821	1.821	1160.059
Port-Bouet	896.124	305.849	1033.649	55.760	55.760	1181.863
Treichville	950.254	220.239	1001.399	189.344	189.344	1152.663
Yopougon	1143.794	188.827	1143.143	1174.250	286.681	2378.507

Table E. Descriptive statistics. Betweenness centrality based on the mobility network.

	Betweenness centrality					
**Commune**	**Mean**	**STD**	**Median**	**Mode**	**Min**	**Max**
Abobo	2642.070	2259.401	1876.346	283.580	283.580	10144.262
Adjame	7693.997	8454.466	3099.174	49.721	49.721	35592.771
Attecoube	4076.686	5433.026	2104.138	965.325	965.325	21049.299
Cocody	904.282	795.863	737.297	0.000	0.000	3795.2082
Koumassi	1431.543	1029.157	1435.936	78.938	78.938	3303.143
Marcory	497.009	819.316	203.666	0.000	0.000	3254.942
Plateau	845.653	962.073	583.866	0.016	0.016	3913.121
Port-Bouet	861.108	1212.989	243.059	0.879	0.879	4312.045
Treichville	1067.158	1365.724	406.400	4.147	4.147	4875.294
Yopougon	2021.051	2263.924	1322.205	0.394	0.394	11101.363

Table F. Descriptive statistics. Effective size based on the communication network.

	**Effective size**					
**Commune**	**Mean**	**STD**	**Median**	**Mode**	**Min**	**Max**
Abobo	624.5552	39.4500	629.4597	472.3369	472.3369	680.0668
Adjame	609.0047	61.9073	621.2195	392.8463	392.8463	672.6013
Attecoube	619.9858	20.8695	618.6310	579.4393	579.4393	647.4868
Cocody	560.8717	119.0959	595.6323	19.4635	19.4635	660.9705
Koumassi	654.2057	14.3398	654.5719	631.0656	631.0656	677.1960
Marcory	573.9489	145.8651	626.9734	11.4110	11.4110	663.8848
Plateau	427.7789	205.7866	546.9742	26.6545	26.6545	646.7823
Port-Bouet	607.6660	111.2927	648.1647	185.6975	185.6975	681.6141
Treichville	612.7983	81.1506	641.0399	283.7057	283.7057	656.3072
Yopougon	599.4598	75.3283	621.9715	219.1734	219.1734	693.3558

Table G. Descriptive statistics. Effective size based on the mobility network.

	**Effective size**					
**Commune**	**Mean**	**STD**	**Median**	**Mode**	**Min**	**Max**
Abobo	369.0861	75.8249	375.5944	184.2824	184.2824	542.8057
Adjame	474.5761	115.4581	435.3778	210.9621	210.9621	722.4065
Attecoube	412.8434	95.8663	398.4168	290.8184	290.8184	622.3746
Cocody	319.6934	88.6299	336.8421	2.0098	2.0098	437.3385
Koumassi	366.3059	72.6580	394.8219	235.1907	235.1907	464.3018
Marcory	256.2028	102.3382	251.9341	3.3112	3.3112	425.9249
Plateau	276.8271	156.1693	361.1846	7.6797	7.6797	453.7765
Port-Bouet	253.2924	104.8842	243.6615	31.4159	31.4159	444.7033
Treichville	331.5060	101.2939	323.2903	66.6192	66.6192	502.7404
Yopougon	350.8463	102.1479	371.5441	38.0114	38.0114	552.2725

Table H. Descriptive statistics. Efficiency based on the communication network.

	**Efficiency**					
**Commune**	**Mean**	**STD**	**Median**	**Mode**	**Min**	**Max**
Abobo	0.5206	0.0292	0.5241	0.4129	0.4129	0.5625
Adjame	0.5090	0.0435	0.5175	0.3637	0.3637	0.5568
Attecoube	0.5160	0.0164	0.5147	0.4857	0.4857	0.5387
Cocody	0.4761	0.0759	0.4977	0.1436	0.1436	0.5463
Koumassi	0.5513	0.0177	0.5503	0.5276	0.5276	0.5887
Marcory	0.5521	0.0572	0.5682	0.2853	0.2853	0.5916
Plateau	0.3937	0.1269	0.4622	0.1339	0.1339	0.5354
Port-Bouet	0.5685	0.0244	0.5732	0.4952	0.4952	0.5959
Treichville	0.5496	0.0335	0.5620	0.4659	0.4659	0.5875
Yopougon	0.5019	0.0498	0.5146	0.3011	0.3011	0.5725

Table I. Descriptive statistics. Efficiency based on the mobility network.

	Efficiency					
**Commune**	**Mean**	**STD**	**Median**	**Mode**	**Min**	**Max**
Abobo	0.88066	0.02174	0.88092	0.81613	0.81613	0.91690
Adjame	0.88360	0.03352	0.87763	0.77846	0.77846	0.93334
Attecoube	0.88201	0.01798	0.87884	0.85535	0.85535	0.92204
Cocody	0.84963	0.06088	0.86694	0.50245	0.50245	0.88889
Koumassi	0.90097	0.00890	0.90292	0.88418	0.88418	0.92004
Marcory	0.88453	0.05031	0.89827	0.66224	0.66224	0.92348
Plateau	0.81130	0.08416	0.86089	0.63997	0.63997	0.89276
Port-Bouet	0.91572	0.01453	0.91819	0.87266	0.87266	0.93362
Treichville	0.89355	0.03030	0.90023	0.78375	0.78375	0.92374
Yopougon	0.88051	0.03786	0.89164	0.66687	0.66687	0.92546

Table J. Descriptive statistics. Local clustering coefficient based on the communication network.

	Local clustering coefficient					
**Commune**	**Mean**	**STD**	**Median**	**Mode**	**Min**	**Max**
Abobo	0.9458	0.0042	0.9448	0.9400	0.9400	0.9638
Adjame	0.9460	0.0070	0.9445	0.9387	0.9387	0.9676
Attecoube	0.9451	0.0023	0.9451	0.9413	0.9413	0.9503
Cocody	0.9483	0.0114	0.9450	0.9381	0.9381	0.9980
Koumassi	0.9465	0.0038	0.9459	0.9413	0.9413	0.9566
Marcory	0.9582	0.0128	0.9546	0.9411	0.9411	0.9923
Plateau	0.9610	0.0200	0.9498	0.9399	0.9399	0.9965
Port-Bouet	0.9565	0.0112	0.9540	0.9413	0.9413	0.9867
Treichville	0.9538	0.0098	0.9530	0.9405	0.9405	0.9809
Yopougon	0.9457	0.0078	0.9437	0.9377	0.9377	0.9844

Table K. Descriptive statistics. Local clustering coefficient based on the mobility network.

	Local clustering coefficient					
**Commune**	**Mean**	**STD**	**Median**	**Mode**	**Min**	**Max**
Abobo	0.66559	0.09156	0.66483	0.46873	0.46873	0.86787
Adjame	0.55492	0.12910	0.57877	0.32386	0.32386	0.89550
Attecoube	0.62562	0.10985	0.64987	0.39461	0.39461	0.76729
Cocody	0.75589	0.08795	0.74844	0.60690	0.60690	1.00000
Koumassi	0.69004	0.07901	0.65119	0.57454	0.57454	0.83479
Marcory	0.80526	0.08263	0.82275	0.62668	0.62668	1.00000
Plateau	0.77635	0.13031	0.72754	0.58432	0.58432	0.95072
Port-Bouet	0.77236	0.08219	0.80957	0.57527	0.57527	0.85397
Treichville	0.73730	0.09869	0.76769	0.52780	0.52780	0.86499
Yopougon	0.68755	0.10680	0.67569	0.45786	0.45786	0.96742

Table L. Linear ordinary least-squares (OLS) of districts' economic well-being, based on districts' positions in the communication network. For ease of interpretation, both efficiency and local clustering coefficient have been standardized. Robust standard errors of parameter estimates are within parentheses. Note: $* = p < 0.05, **p < 0.01, ***p < 0.001$.

	Revenue budget per person		Capital budget per person		Percentage of land covered by unshared houses		Percentage of land covered by shared houses		Percentage of land covered by slums		Informal settlement land use	
	Mod. I	Mod. II	Mod. I	Mod. II	Mod. I	Mod. II	Mod. I	Mod. II	Mod. I	Mod. II	Mod. I	Mod. II
**Open structures:**												
Betweenness centr.	-177.04**		-25.85**		-0.09		-0.03		0.15*		0.15*	
	(41.35)		(6.65)		(0.05)		(0.01)		(0.04)		(0.04)	
Efficiency	-35,564.07**	-35,453.44**	-5,432.05**	-5,420.03***	-8.21	-8.44	-5.35**	-5.37**	3.17	3.42	3.17	3.42
	(7,319.88)	(6,307.11)	(1,027.51)	(882.36)	(6.70)	(6.93)	(1.44)	(1.40)	(5.91)	(6.41)	(5.91)	(6.41)
**Closed structures:**												
Local clust. coeff.		25,756.8**		3,740.14		12.47		3.61		-20.50*		-20.50*
		(5,441.82)		(877.93)		(7.44)		(1.86)		(6.54)		(6.54)
Constant	202,008.6**	21,416.8**	30,674.66**	4,308.9**	136.43*	39.25**	35.99*	9.29**	-117.19*	38.60*	-117.19*	38.6088
No. observations	10	10	10	10	10	10	10	10	10	10	10	10
R^2	0.88	0.90	0.87	0.89	0.45	0.43	0.64	0.64	0.54	0.52	0.54	0.52
Root MSE	19,994	17,754	3,086.3	2,812.5	21.04	21.52	6.00	6.00	22.81	23.41	22.81	23.41
F(2, 7)	21.04**	33.32***	23.22***	35.81***	5.17*	5.02*	52.34***	56.13***	9.19*	9.09*	9.19*	9.09*

Table M. Linear ordinary least-squares (OLS) and negative binomial models of districts' democratic participation, public safety and security, based on districts' positions in the communication network. For ease of interpretation, both efficiency and local clustering coefficient have been standardized. Robust standard errors of parameter estimates are within parentheses. Note: $* = p < 0.05, ** p < 0.01, ***p < 0.001$.

	Democratic participation		Security (land coverage)		Stolen cars (2009)	
	Mod. I	Mod. II	Mod. I	Mod. II	Mod. I	Mod. II
**Open structures:**						
Betweenness centr.	0.0002		-0.00003*		0.001	
	(0.0002)		(9.80e-06)		(0.009)	
Efficiency	0.02	0.02	-0.001	-0.001	0.001	-0.22
	(0.02)	(0.02)	(0.002)	(0.002)	(1.85)	(0.67)
**Closed structures:**						
Local clust. coeff.		-0.02		0.004*		0.23
		(0.02)		(0.002)		(0.20)
Constant	0.09	0.26***	0.05**	0.01***	3.09	3.71***
No. observations	10	10	10	10	10	10
R^2	0.20	0.19	0.49	0.44		
Root MSE	0.07	0.076	0.006	0.01		
$F(2,7)$	2.64	2.63	9.83**	8.73*		
$PseudoR^2$					0.0002	0.002
Wald $\chi^2(2)$					0.07	1.30
Log pseudolikelihood					-41.82	-41.73

Table N. Linear ordinary least-squares (OLS) of districts' economic well-being, based on districts' positions in the mobility network. For ease of interpretation, both efficiency and local clustering coefficient have been standardized. Robust standard errors of parameter estimates are within parentheses. Note: $* = p < 0.05, ** p < 0.01, *** p < 0.001$.

	Revenue budget per person		Capital budget per person		Percentage of land covered by unshared houses		Percentage of land covered by shared houses		Percentage of land covered by slums		Informal settlement land use	
	Mod. I	Mod. II	Mod. I	Mod. II	Mod. I	Mod. II	Mod. I	Mod. II	Mod. I	Mod. II	Mod. I	Mod. II
**Open structures:**												
Betweenness centr.	-3.09			-0.53		-0.005		0.0003		0.02	0.01	
	(3.03)		(0.43)		(0.003)		(0.001)		(0.004)		(0.004)	
Efficiency	-39,347.06	-38,243.61	-6,054.08*	-5,895.35*	-12.88**	-11.21*	-6.34*	-6.19**	7.24	4.78	7.24	4.78
	(17,260.07)	(16,446.47)	(2,414.17)	(2,302.99)	(3.32)	(4.36)	(1.81)	(1.70)	(7.06)	(5.27)	(7.06)	(5.27)
**Closed structures:**												
Local clust. coeff.		9,350.67		1,450.69		15.30*		0.27		-21.25*		-21.25*
		(7,610.15)		(1,096.96)		(5.67)		(2.67)		(8.40)		(8.49)
Constant	28,229.06	21,416.8	5,469.21*	4,308.9*	51.54***	39.25***	8.51*	9.29**	22.73	38.60**	22.73	38.60**
No. observations	10	10	10	10	10	10	10	10	10	10	10	10
R^2	0.65	0.67	0.69	0.70	0.56	0.68	0.51	0.50	0.38	0.58	0.38	0.58
Root MSE	33,603	32,874	4,821.4	4,729.9	18.76	16.02	7.005	7.05	26.67	21.83	26.67	21.83
$F(2,7)$	2.65	2.91	3.25	3.53	10.73**	9.55*	6.19*	7.19*	2.99	5.99*	2.99	5.99*

Table O. Linear ordinary least-squares (OLS) and negative binomial models of districts' democratic participation, public safety and security, based on districts' positions in the mobility network. For ease of interpretation, both efficiency and local clustering coefficient have been standardized. Robust standard errors of parameter estimates are within parentheses. Note: $* = p < 0.05, **p < 0.01, ***p < 0.001$.

	Democratic participation		Security (land coverage)		Stolen cars (2009)	
	Mod. I	Mod. II	Mod. I	Mod. II	Mod. I	Mod. II
**Open structures:**						
Betweenness centr.	0.00002**		-1.48e-06		-0.00006	
	(5.89e-06)		(9.98e-07)		(0.00008)	
Efficiency	0.02	0.02	-0.002	-0.002	-0.21	-0.22
	(0.02)	(0.02)	(0.002)	(0.001)	(0.65)	(0.67)
**Closed structures:**						
Local clust. coeff.		-0.04**		0.004		0.23
		(0.01)		(0.002)		(0.20)
Constant	0.22**	0.26***	0.02***	0.01***	3.86***	3.71***
No. observations	10	10	10	10	10	10
R^2	0.40	0.47	0.38	0.57		
Root MSE	0.06	0.06	0.01	0.005		
$F(2,7)$	10.55**	12.24**	3.18	5.42*		
$PseudoR^2$					0.001	0.002
Wald $\chi^2(2)$					0.64	1.30
Log pseudolikelihood					-41.77	-41.73
